# Metabolomic Analysis of *Stephania tetrandra*–*Astragalus membranaceus* Herbal Pair-Improving Nephrotic Syndrome Identifies Activation of IL-13/STAT6 Signaling Pathway

**DOI:** 10.3390/ph16010088

**Published:** 2023-01-08

**Authors:** Baiyang Xu, Mengxue Yao, Zilu Liu, Shanshan Zhang, Bin Wang, Yanquan Han, Jiarong Gao, Deling Wu, Xiaoli Wang

**Affiliations:** 1 Key Laboratory of Chinese Medicinal Formula of Anhui Province, Anhui University of Chinese Medicine, Hefei 230031, China; 2 The First Affiliated Hospital, Anhui University of Chinese Medicine, Hefei 230031, China; 3 Anhui Province Key Laboratory of Traditional Chinese Medicine Decoction Pieces of New Manufacturing Technology, Bozhou 236800, China; 4 Anhui Province Key Laboratory of Research & Development of Chinese Medicine, Hefei 230012, China

**Keywords:** *Stephania tetrandra*, *Astragalus membranaceus*, herbal pair, nephrotic syndrome, untargeted metabolomics, IL-13/STAT6 signal pathway

## Abstract

The *Stephania tetrandra*–*Astragalus membranaceus* herbal pair (FH) is a classic herbal pair widely used in the treatment of nephrotic syndrome (NS). The effects of *Stephania tetrandra* (FJ) and *Astragalus membranaceus* (HQ) on NS have been reported, but the mechanism of their combination on the improvement of NS are still unclear. The NS model was established by injecting adriamycin into the tail vein. FH intervention reduced the levels of serum triglyceride, total cholesterol, interleukin-6 (IL-6), blood urea nitrogen (BUN), urinary protein, and the gene expression levels of aquaporin 2 (AQP2) and arginine vasopressin (AVP) in NS rats. In addition, FH improved kidney injury in NS rats by inhibiting the expression of interleukin 13 (IL-13), phospho-signal transducers, and activators of transcription 6 (p-STAT6), Bax, cleaved-caspase3, while promoting the expression of Bcl-2. By comprehensive comparison of multiple indexes, the effects of FH on lipid metabolism, glomerular filtration rate, and inflammation were superior to that of FJ and HQ. Metabonomic studies showed that, compared with FJ and HQ, FH intervention significantly regulated tricarboxylic acid (TCA) cycle, cysteine and methionine metabolism, and alanine, aspartic acid and glutamic acid metabolism. Pearson correlation analysis showed that succinic acid and L-aspartic acid were negatively correlated with urinary protein, cystatin C (Cys C) and BUN (*p* < 0.05). In summary, FH could reduce renal injury and improve NS through inhibiting the IL-13/STAT6 signal pathway, regulating endogenous metabolic pathways, such as TCA cycle, and inhibiting the expression of AQP2 and AVP genes. This study provides a comprehensive strategy to reveal the mechanism of FH on the treatment of NS, and also provides a reasonable way to clarify the compatibility of traditional Chinese medicine.

## 1. Introduction

Nephrotic syndrome (NS) is a common urinary disease, clinically manifested by proteinuria, hypoproteinemia, edema, hyperlipidemia, chronic inflammation, and renal injury [1]. The pathogenesis of NS is complex, and the damage of glomerular filtration barrier is the most important pathophysiological basis of NS [2]. Hyperlipidemia in NS is characterized by low density of lipoprotein and elevated serum total cholesterol. This process is associated with abnormal lipid metabolism. Dyslipidemia, especially the abnormal increase in serum cholesterol level, is a key factor of glomerular injury or sclerosis. However, high fat is not a single factor in kidney injury, inflammation has also been shown to promote glomerular disease. Studies have revealed that inflammatory factors, such as interleukin-1 (IL-1), interleukin-6 (IL-6), tumor necrosis factor-α (TNF-α), and transforming growth factor-β (TGF-β), can aggravate glomerular injury [3]. Moreover, there was evidence that interleukin-13 (IL-13)/ signal transducer and activator of transcription 6 (STAT6) signal pathway mediates inflammation and apoptosis to aggravate renal injury. The edema of NS has been proved to be related to the abnormal expression of aquaporin (AQP). It was found that AQP2 is involved in the reabsorption of water in the kidney, and its high expression may lead to the retention of water and sodium [4]. The expression of AQP2 is regulated by arginine vasopressin (AVP), which changes the water permeability of the principal cells of the collecting duct by regulating the expression of AQP2. Compared with modern drugs, traditional Chinese medicine (TCM) has unique advantages in the treatment of NS, especially in reducing proteinuria and the side effects of immunosuppressive drugs. Moreover, compared with hormone therapy, TCM treatment of NS can better reduce the recurrence rate of disease [5].

Fangji Huangqi Tang (FHT) is a classic and practical prescription of TCM. Pharmacological studies have shown that FHT has an important clinical effect on rheumatic kidney deficiency and spleen deficiency and can enhance the immune system [6]. *Stephania tetrandra* (FJ) and *Astragalus membranaceus* (HQ) are both the monarch drug in FHT. The *Stephania tetrandra*–*Astragalus membranaceus* herbal pair (FH) is the classic herbal pair recorded in “Shi Jin Mo Dui Yao”. It also has the effects of beneficial Qi, invigorating the spleen, and reducing edema. The active ingredients in FJ are mainly dibenzyl isoquinoline alkaloids, such as tetrandrine, fangchinoline, and rocinoline, which have analgesic, antitumor, antifibrotic, and anti-inflammatory effects [7,8]. Our previous study found that N-methylfangchinoline, fangchinoline, tetrandrine, tetrandrine-M3, and tetrandrine-M4 have the same structure, which can cooperatively improve kidney injury [9]. The main components of HQ are flavonoids, saponins, and polysaccharides, such as calycosin-7-glucoside, isolicorice, and astragalus IV, which can enhance immune function, improve cardiovascular function, inhibit apoptosis, and have anti-ulcer and anti-inflammatory effects [10,11]. FJ and HQ have the potential to treat NS. FH has a good therapeutic effect on NS. To the best of our knowledge, the synergistic mechanism of its anti-nephrotic syndrome has not been clearly reported.

In recent years, metabolomics emphasizes the holistic approach to study the functional level of organisms, which is consistent with the holistic view of TCM theory [12,13]. According to the published literature and available information, metabolomics has attracted the attention of researchers around the world. Metabolomics has the characteristics of reflecting the overall functional state of organisms and its terminal amplification. Metabolome refers to intermediates and end products of gene expression and metabolism [14]. Thus, the pathological or physiological state of an organism can be determined by detecting temporal changes in their composition and content. In addition, it is possible to find relevant biomarkers for disease diagnosis [15]. Gas chromatography–mass spectrometry (GC-MS) has been widely used for the identification and quantification of metabolites due to its high resolution, reproducibility, sensitivity, and other characteristics in metabolomics research [16]. Existing metabolomics data analysis methods are difficult to show the overall changes in biological metabolism in a more specific way. Therefore, this study introduced the multi-attribute comprehensive index method to analyze and compare the overall metabolic level in a deeper way.

The multi-attribute comprehensive index method is a comprehensive evaluation method that standardizes the indexes of different categories, different properties, and different units of measurement, and finally transforms them into a dimensionless relative evaluation value, which can reflect the relative level and overall change [17]. In this paper, the frequency of relevant effect indexes in the literature was used to comprehensively determine the weight coefficients and variable importance in projection (VIP), and partial least-squares discriminant analysis (PLS-DA) was used to score the metabolomics and efficacy indexes of FH, FJ, and HQ, which provided an objective theoretical basis for determining the weight coefficients. The effects of FH drug pair and single drug on metabolism and overall efficacy of NS rats were evaluated. In addition, in order to further explore the relationship between changes in endogenous metabolism and drug efficacy, Pearson correlation analysis was also adopted in this paper. This method can screen out relevant variables more quickly, which is also of great help in finding endogenous biomarkers.

**Figure 1:**
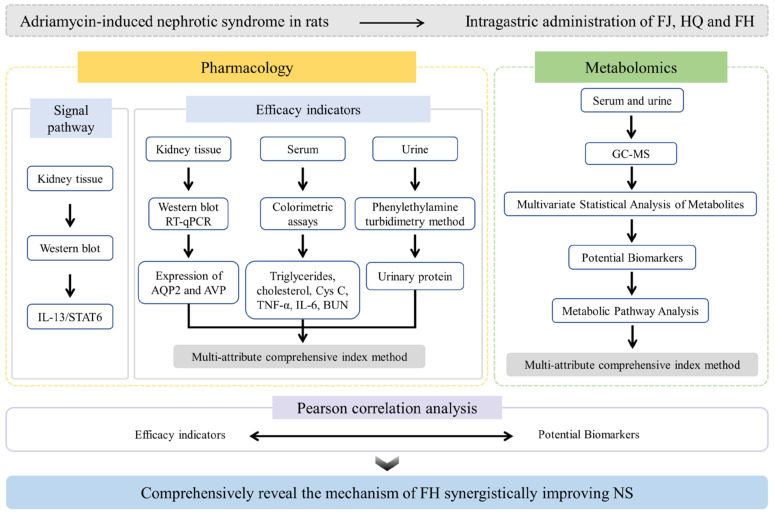
The study design of this work to reveal the mechanism of synergistic improvement of nephrotic syndrome by the *Stephania tetrandra*–*Astragalus membranaceus* herbal pair.

In this study, the rat model of NS was established by intravenous injection of adriamycin. FJ, HQ, and FH were intragastrically administered continuously. The integrated strategy of pharmacodynamics and metabolomics was used to explore the effects of *Stephania tetrandra*–*Astragalus membranaceus* herbal pair on the overall efficacy, endogenous substance metabolism, and signal pathway in NS rats before and after compatibility. The multi-attribute comprehensive index method was used to compare the overall synergistic effect of *Stephania tetrandra*–*Astragalus membranaceus* herbal pair. Pearson correlation analysis was used to analyze the correlation between potential biomarkers and efficacy indicators to reveal the mechanism of synergistic improvement of NS by FH. The study design for this work was shown in Figure 1.

## 2. Results

### 2.1. Efficacy Evaluation of FJ, HQ, and FH in Rats with NS

#### 2.1.1. General Morphological Observation

Rats in the control (K) group had normal diet and activities, smooth and bright hair, normal urine and stool, and normal weight increasing with time. Rats in the model (M) group showed matte hair, testiness disposition, slow movement, dispirited spirit, and both food intaking and urine outputting reduced.

#### 2.1.2. Histopathology Analysis

**Figure 2:**
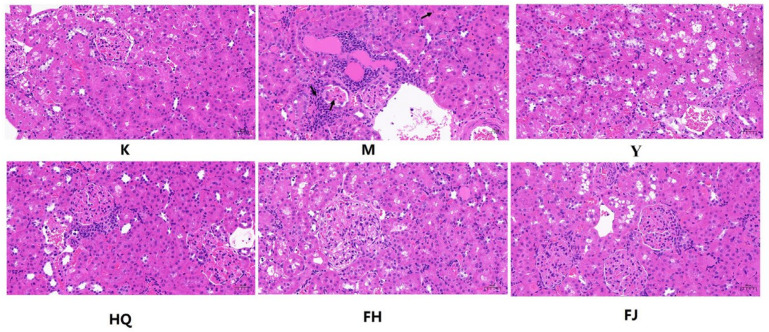
Hematoxylin-eosin (HE) staining was used to evaluate the pathological changes in renal tissue (Light microscopy, ×200): (K) control; (M) model; (Y) Huangkui capsule (Y, 1 g/kg); (FJ) Fang Ji (FJ, 4 g/kg); (HQ) Huang Qi (HQ, 5 g/kg); (FH) Fangji Huangqi herb couples (FH, 9 g/kg). Note: the black arrow indicates the location of the lesion.

Paraffin-embedded sections and HE staining were performed to evaluate the overall pathological changes of kidney tissues (Figure 2). Kidneys of the M group animals exhibited glomerular volume increasing, basal membrane hyperplasia, renal tubular epithelial cell atrophy, and severe inflammatory cell infiltration. FJ, HQ, and FH treatments significantly improved these changes, and FH had the best effect.

#### 2.1.3. Blood Urea Nitrogen, Triglycerides, Cholesterol, Cys C, TNF-α, IL-6, Urinary Protein, AQP2 Gene, AQP2 Protein, AVP Gene, and AVP Protein Detection

**Figure 3:**
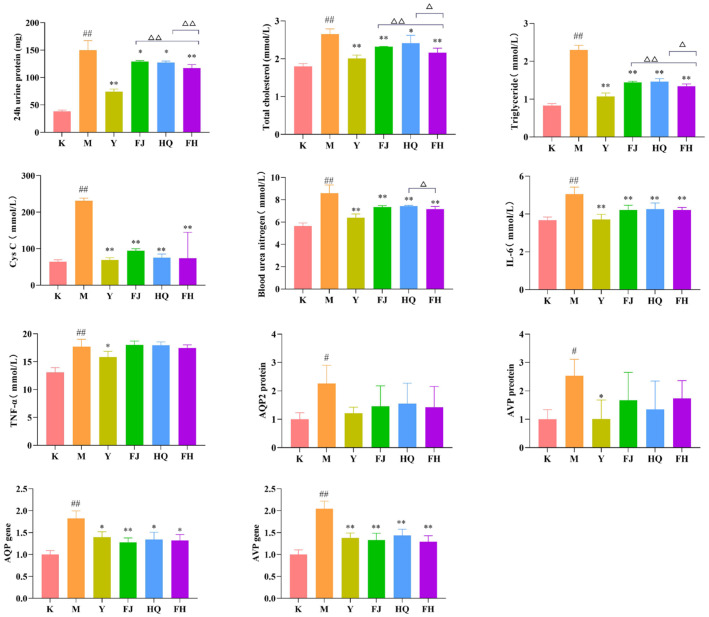
The 24 h urinary protein quantification, cholesterol, triglyceride, Cys C, BUN, IL-6, TNF-α levels (*n* = 6), and AQP2 and AVP protein and gene expression levels (*n* = 3) in each group. Compared with the K group, ^#^ *p* < 0.05, ^##^ *p* < 0.01; compared with the M group, * *p* < 0.05, ** *p* < 0.01; compared with FH group, ^△^ *p* < 0.05, ^△△^ *p* < 0.01.

As shown in Figure 3, the content of serum total cholesterol, triglyceride, blood urea nitrogen (BUN), IL-6, Cystatin C (Cys C), TNF-α, urinary protein, and the expression of AQP2 gene, AVP gene, AQP2 protein, and AVP protein in the M group were dramatically higher than those in group K, indicating that group M showed obvious symptoms of NS. After HQ, FJ, and FH treatment, these changes were significantly improved. Moreover, the effects of FH on serum total cholesterol, triglyceride, BUN, and urinary protein were greater than those of FJ or HQ alone.

### 2.2. Effects of FJ, HQ, and FH on Gene Expression of Apoptosis Factors and IL-13/STAT6 Signal Pathway in Renal Tissue

**Figure 4:**
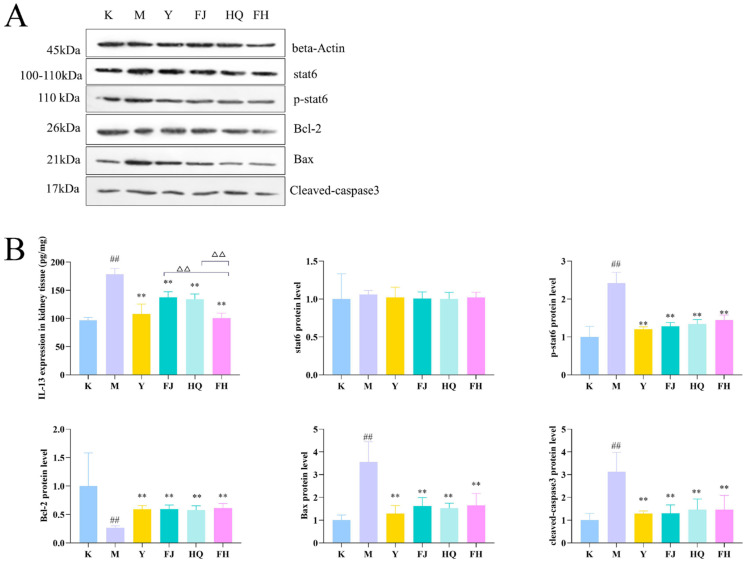
Western blot analysis of the effects of Y, FJ, HQ, and FH on the expression of p-STAT6, Bcl-2, Bax, and cleaved-caspase3 (**A**). Relative quantitative data for p-STAT6, Bcl-2, Bax, and cleaved-caspase3 (*n* = 3), and levels of IL-13 in kidney tissue (*n* = 6) (**B**). Compared with the K group, ^##^ *p* < 0.01; compared with the M group, ** *p* < 0.01; compared with the FH group, ^△△^ *p* < 0.01.

As shown in Figure 4, the level of IL-13 and the protein expression of phospho-STAT6 (p-STAT6), Bax, and cleaved-caspase3 in renal tissue of group M were significantly higher than those of group K, while Bcl-2 was significantly decreased. The trend of the above indexes in the administration groups were opposite to that in the M group. In addition, the effect of FH on IL-13 was significantly better than that of FJ and HQ.

### 2.3. Multi-Attribute Comprehensive Index Analysis of Efficacy

**Figure 5:**
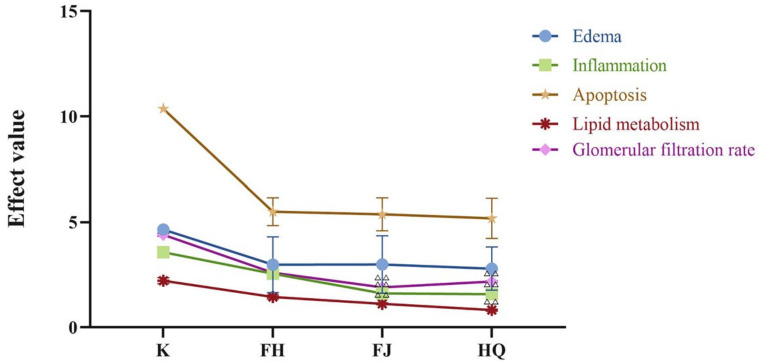
Effects of FJ, HQ, and FH on lipid metabolism, glomerular filtration rate, edema, and inflammatory cell apoptosis in rats with nephrotic syndrome. Note: Compared with FH group, ^△△^ *p* < 0.01.

To further analyze the differences of FJ, HQ, and FH, the effects of the three on lipid metabolism, glomerular filtration rate, edema, inflammation, and apoptosis were analyzed by multi-attribute comprehensive index method. According to the literature review, the number of studies related to NS and each index is listed below: total cholesterol (253), triglyceride (64), urinary protein (1409), BUN (49), Cys C (20), AQP2 (13), AVP (6), IL-13 (18), TNF-α (59), p-STAT6 (1), IL-6 (54), Bcl-2 (10), Bax (12), STAT6 (1), cleaved-caspase3 (3). The weight coefficient and VIP value of each indicator are shown in Table S1 and Figure S1 in the Supplementary Materials. The total effects of lipid metabolism, glomerular filtration rate, edema, inflammation, and apoptosis are shown in Figure 5. Compared with FJ and HQ, the effect values of lipid metabolism, glomerular filtration rate, inflammation, and apoptosis of FH were closer to those of the K group, and the effect values of lipid metabolism, glomerular filtration rate, and inflammation were significantly higher than those of FJ and HQ.

### 2.4. GC–MS Analysis

#### 2.4.1. Multivariate Statistical Analysis of Metabolites

**Figure 6:**
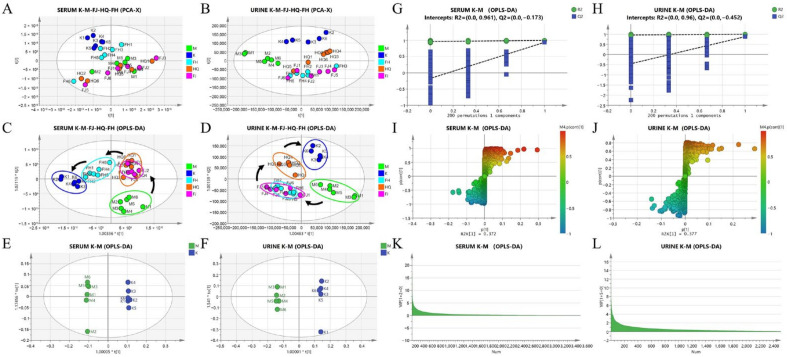
Metabolic profile analysis of serum and urine samples in each group. Note: PCA score plots for serum (**A**) and urine (**B**) samples in each group; OPLS-DA score plots for serum (**C**) and urine (**D**) samples in each group; OPLS-DA score plots of serum (**E**) and urine (**F**) samples of K group and M group; permutation plots of serum (**G**) and urine (**H**) samples of K group and M group; S-plot of serum (**I**) and urine (**J**) samples of K group and M group; VIP plots of serum (**K**) and urine (**L**) samples from K and M groups.

The total ion chromatograms (TIC) of serum and urine metabolites in each group were shown in Figure S2. Principal component analysis (PCA) and orthogonal partial least-squares discriminant analysis (OPLS-DA) were utilized to analyze the metabolic profiles of serum and urine in each group. The score plots showed that there was a great difference in the distribution of metabolites between the K group and the M group, while the distribution of metabolites in each treatment group tended towards that in the K group (Figure 6A–D).

In order to screen differential metabolites, OPLS-DA model was used to maximize the discriminant ability of serum and urine metabolites between the K group and the M group. The permutation chart of serum and urine samples showed that the values of blue *Q*^2^ and green *R*^2^ on the left side of the quadrant were lower than those of the original point on the right, and the blue line of regression of *Q*^2^ intersected with the longitudinal axis below zero, indicating the effectiveness of the model. The S-plot of serum and urine samples showed that the spots close to p values of 1 and -1 were denser, indicating that there may be more potential differential metabolites. The key variables are filtered according to the VIP value, and the threshold is 1.0 (Figure 6E–L).

#### 2.4.2. Potential Biomarkers Screened by Qualitative Metabolomics Analysis

The potential biomarkers screened by metabolomics technology are expected to be an important reference index for disease diagnosis. According to the criteria of VIP > 1, *p* < 0.05 and NIST match factor > 700, 18 metabolites (12 in serum and 6 in urine) associated with NS were identified in group M compared with group K (Table 1). These potential biomarkers were mainly associated with organic acids, amino acid, fatty acids, carbohydrates, and their products.

**Figure 7:**
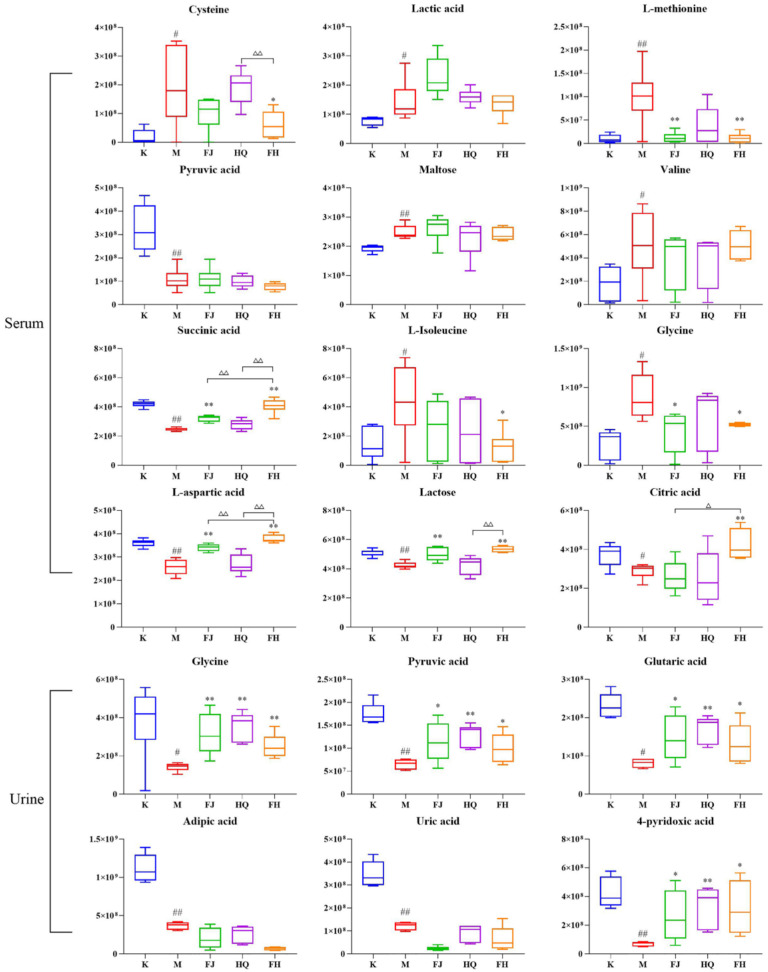
Expression levels of differentially expressed metabolites in serum and urine. Note: Compared with the K group, ^#^ *p* < 0.05, ^##^ *p* < 0.01. Compared with the M group, * *p* < 0.05 and ** *p* < 0.01. Compared with FH group, ^△^ *p* < 0.05, ^△△^ *p* < 0.01.

Compared with the K group, seven metabolites (cysteine, lactic acid, L-methionine, maltose, valine, L-isoleucine, and glycine) in the serum of the M group were significantly increased, while five metabolites (pyruvic acid, succinic acid, L-aspartic acid, lactose, and citric acid) were significantly decreased; six metabolites (glycine, pyruvic acid, glutaric acid, adipic acid, uric acid, and 4-pyridoxic acid) in urine of the M group decreased significantly. The intervention of FJ, HQ, and FH reversed the changes of most differential metabolites, and all metabolites regulated by FJ and HQ were also regulated by FH. However, not all metabolites regulated by FH were regulated by FJ or HQ. FH significantly regulated cysteine, L-methionine, succinic acid, L-isoleucine, L-aspartic acid, lactose, and citric acid in serum and glycine, pyruvic acid, glutaric acid, and 4-pyridoxic acid in urine. Among them, the regulatory effect of FH on serum cysteine, succinic acid, L-aspartic acid, and citric acid was significantly better than that of FJ or HQ (Figure 7).

#### 2.4.3. Enrichment and Analysis of Metabolic Pathway

**Figure 8:**
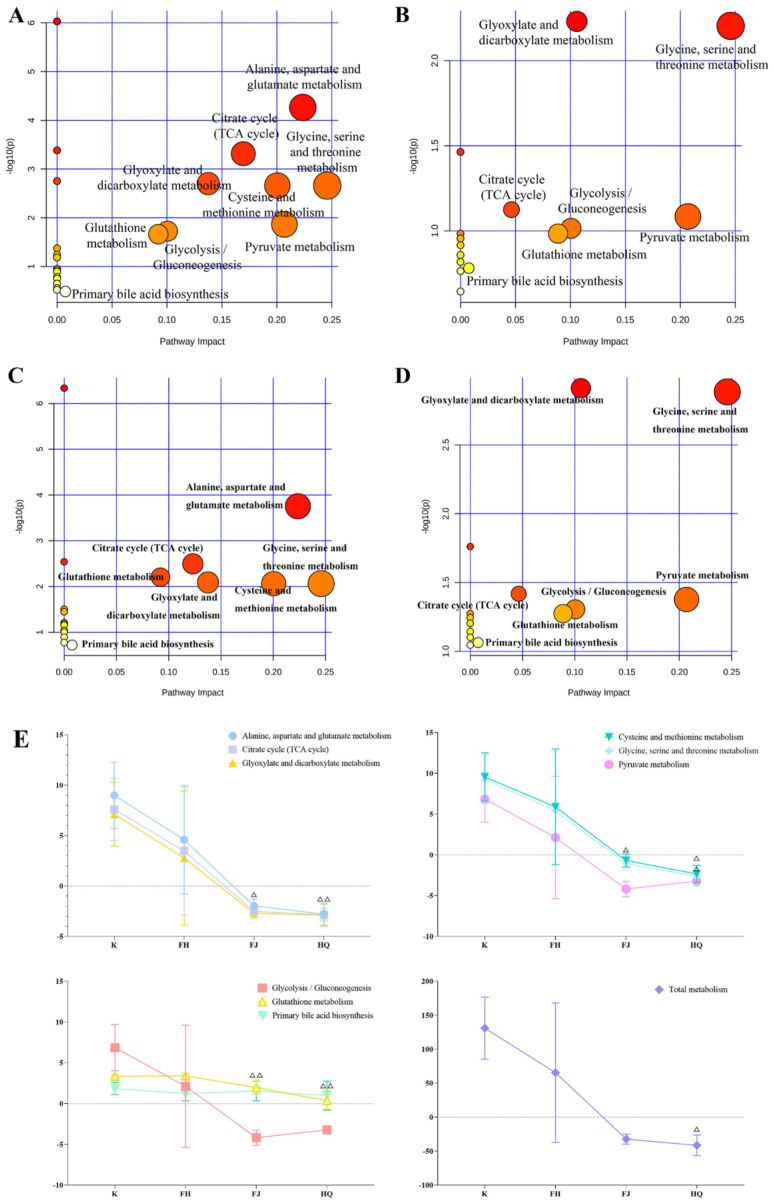
Metabolic pathways of blood and urine samples in rats with nephrotic syndrome and their effects after administration. Note: (**A**) serum sample metabolic pathways of NS rats; (**B**) urine sample metabolic pathways of NS rats; (**C**) serum metabolic pathways regulated by FH; (**D**) urine metabolic pathways regulated by FH; (**E**) effect values of metabolic pathways in serum and urine samples of K group and drug administration group. Compared with FH group, ^△^ *p* < 0.05, ^△△^ *p* < 0.01.

In order to explore the potential pathways influenced by NS and the metabolic pathways regulated by FH, the differential endogenous metabolites and FH-regulated metabolites in Table 1 were introduced into MetaboAnalyst4.02 (Xia Lab @ McGill, Quebec, Canada). As shown in Figure 8A–D, NS was associated with nine key metabolic pathways (for ease of description, nine metabolic pathways are numbered from MP1~MP9), including: citrate cycle (TCA cycle) [MP1]; alanine, aspartate and glutamate metabolism [MP2]; glyoxylate and dicarboxylate metabolism [MP3]; cysteine and methionine metabolism [MP4]; glycine, serine and threonine metabolism [MP5]; pyruvate metabolism [MP6]; glycolysis / gluconeogenesis [MP7]; glutathione metabolism [MP8]; primary bile acid biosynthesis [MP9]. In addition, FH regulated all of these nine key metabolic pathways.

#### 2.4.4. Multi-Attribute Comprehensive Index Analysis of Metabolism

In order to further explore the effects of FH, FJ, and HQ on metabolic pathways in rats with NS, the related metabolites and metabolic pathways of each group were scored by multi-attribute comprehensive index method. Firstly, the number of studies in the literature with “nephrotic syndrome” and various metabolites and metabolic pathways as keywords in the past five years is as follows: cysteine, citric acid, glycine, pyruvic acid, succinic acid, lactic acid, L-methionine, L-aspartic acid were reported, respectively in 17, 5, 5, 4, 4, 3, 2, 0 articles; MP1, MP2, MP3, MP4, MP5, MP6, MP7, MP8, MP9 were reported, respectively in 5, 4, 3, 2, 1, 5, 1, 6, 0 articles. Then, the VIP values of each metabolic index in serum and urine samples were shown in Figure S3. Combined with the number of studies in the ltierature and VIP value, the weight of each metabolic index was shown in Table S2. As shown in Figure 8E, the effect values of each metabolic pathway and total metabolism in the FH group were the closest to those in the K group. The effect values of MP2, MP4, and MP8 in the FH group were significantly higher than those in the FJ group and the HQ group. In addition, the effect values of MP1, MP5, and total metabolism in the FH group were significantly higher than those in the HQ group.

### 2.5. Pearson Correlation between Various Endogenous Metabolites and Efficacy Indicators

**Figure 9:**
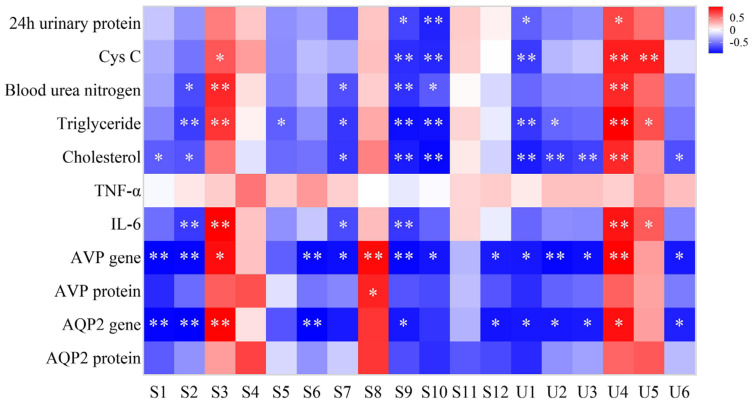
Association plot of rat serum and urine metabolites and efficacy indicators according to Pearson correlation coefficient. The degree of correlation is shown on a color scale from significant negative correlation (blue) to significant positive correlation (red). S1~S12 are cysteine, lactic acid, L-methionine, L-isoleucine, valine, glycine, citric acid, pyruvic acid, succinic acid, respectively. L-aspartic acid, lactose, and maltose; U1~U6 are glycine, pyruvic acid, glutaric acid, adipic acid, uric acid, and 4-pyridoxic acid, respectively. * and ** indicate significant correlation at 0.05 and 0.01 levels (double tails), respectively.

The correlation map of metabolites and efficacy indicators of NS was generated based on Pearson correlation coefficient. The correlation heat map (Figure 9) showed that S1, S2, U2, U3, U6 (cysteine, lactic acid, pyruvic acid, glutaric acid, 4-pyridoxic acid) were negatively correlated with cholesterol, AVP gene, and AQP2 gene. The metabolites of S3, U4, and U5 (L-methionine, adipic acid, uric acid) were positively correlated with Cys C, triglyceride and IL-6. S7 (citric acid) were negatively correlated with BUN, triglyceride, cholesterol, IL-6, and AVP gene. S9, S10, U1 (succinic acid, L-aspartic acid, glycine) were negatively correlated with urinary protein, Cys C, triglyceride, cholesterol, and AVP gene.

## 3. Discussion

FJ and HQ are the classic herbal pair recorded in “Shi Jin Mo Dui Yao”, and they are the monarch medicine in FHT. FHT is a classic Chinese medicine prescription against NS. Pharmacological studies have shown that FH has a good effect on the improvement of NS [18], but its specific regulatory mechanism and compatibility changes are still unclear.

The rat model of NS was established by injection of adriamycin. Injection of adriamycin in rats resulted in decreased food intake and urine output, hyperplasia of glomerular basement membrane, enlargement of glomerular basement membrane, atrophy of renal tubular epithelium, and severe inflammatory cell infiltration. In addition, the significant increase of proteinuria, BUN, and Cys C in the M group also indicated impaired renal function [19]. The results showed that the serum levels of cholesterol, triglyceride, TNF-α, and IL-6 in rats with nephrotic syndrome were increased and the gene and protein expressions of AQP2 and AVP were up-regulated in rats with adriamycin injected into caudal vein. Huangkui capsule has been proved to be effective in improving adriamycin-induced nephropathy and is widely used in clinical treatment [20]. In this study, the renal tissue of rats in the Huangkui capsule group (Y) was significantly better than that in the M group. Huangkui capsule also improved the levels of urinary protein, cholesterol, triglyceride, CysC, BUN, IL-6, and TNF- α in NS rats. It was suggested that Huangkui capsule can protect the kidney from adriamycin-induced NS in this study, and it could also support the therapeutic effect of other drug groups. FH also had a significant effect on these pathological changes in NS rats. These results suggested that FH may regulate lipid metabolism, inflammation, and edema in NS.

### 3.1. Effect of FH on Lipid Metabolism in NS

Dyslipidemia is one of the main features of NS. Dyslipidemia can lead to the disturbance of lipid metabolism in the body and increase the risk of myocardial infarction [2]. Modern scholars have found that dyslipidemia is related to dysregulation of lipid metabolism. Clinical studies showed that the serum cholesterol and triglyceride levels of NS patients were elevated. As the main carrier of blood lipids, lipoproteins are involved in the main pathway of lipid production and transportation in vivo. Therefore, the pathogenesis of dyslipidemia usually includes metabolic abnormalities in triglyceride, very low-density lipoprotein, and fatty acid metabolism, as well as cholesterol metabolism [21]. In the present study, serum cholesterol and triglyceride in M group were significantly increased. However, the levels of the FJ, HQ, and FH groups decreased significantly, and the regulatory effect of FH was more significant than that of single drug. In the subsequent efficacy multi-attribute comprehensive index analysis, the effect value of FH on lipid metabolism was significantly higher than that of FJ and HQ. All these suggested that FH may improve dyslipidemia in NS by regulating lipid metabolism (cholesterol, triglyceride).

### 3.2. Effect of FH on Inflammation in NS

Many studies have shown that renal inflammation and apoptosis are usually associated with the development and progression of pathological features of NS [22]. Abnormal expression of proinflammatory cytokines, such as IL-6 and TNF-α, in the kidney recruits associated inflammatory cells and causes inflammation in the kidney tissue. In addition, pro-inflammatory cytokines can trigger cell apoptosis and promote the synthesis of other inflammatory molecules, thus causing inflammatory response [23]. Apoptosis leads to the loss of resident kidney cells and subsequent renal injury induced by environmental and intrinsic stimuli. STAT6 is a key transcription factor associated with IL-13 [24]. When activated and phosphorylated, STAT6 regulates gene expression in different cells in the nucleus and mediates many pathological features of inflammatory responses in animal models [25]. Studies have shown that the significantly increased expression of STAT6mRNA may be connected with the early course of NS [26]. IL-13 promotes STAT6 phosphorylation and nuclear translocation, and may participate in inflammation and apoptosis in renal injury [27]. This suggests that the IL-13/STAT6 signal pathway may play a key role in NS.

In this study, we observed that the concentration of proinflammatory cytokines in serum of NS rats increased significantly, the IL-13/STAT6 signal pathway was overexpressed in kidney tissue, the expression of pro-apoptotic factors increased, and the expression of anti-apoptotic factor Bcl-2 decreased. After administration of FH, it was obviously improved. Multi-attribute comprehensive index analysis showed that FH was better than FJ or HQ in the regulation of inflammation and apoptosis in rats with NS. It was reported that tetrandrine, calycosin-7-glucoside, and fangchinoline have pharmacological effects of anti-inflammation, antioxidation, and inhibition of apoptosis [28,29,30], and astragalus IV have been reported to reshape STAT signal transduction and reduce inflammatory response [31]. Some of these components were also detected in FH concentrate, but the mechanism of these components in improving NS remains to be further studied. This study suggested that FH may reduce inflammation and apoptosis in NS rats by regulating the IL-13/STAT6 signal pathway.

### 3.3. Effects of FH on Water-Liquid Metabolism in NS

Edema is one of the main clinical symptoms of NS, which is associated with water and sodium retention [32]. Abundant evidence has shown that fluid retention in renal diseases is closely related to changes in renal AQP expression [33,34]. Some studies have found that the level of AQP2 in kidney and urine of NS patients was significantly increased, and it was more obvious in patients with edema [4]. AQP2 is the most widely distributed AQP subtype in kidney. It is expressed in major cells of the lumen plasma membrane of the collecting duct and intracellular vesicles. AQP2 is a key protein involved in water reabsorption in the kidney and plays a crucial role in maintaining water balance and regulating body fluid volume and osmotic pressure [35]. AQP2 is triggered by AVP and then transferred from the cytoplasm to the luminal vesicle. The density of AQP2 in the lumen membrane increases, thus increasing its permeability to water [36]. As a result, water is absorbed, transported through the bone marrow osmotic gradient to the interstitium, and then transferred back into the bloodstream through the renal vessels. Long-term regulation occurs when AVP levels continue to rise, leading to increased AQP2mRNA levels and AQP2 molecules being cleared in the urine [37]. In this study, the expressions of AVP and AQP2 were significantly increased in renal tissues of rats with NS. This trend was consistent with NS patients with edema. FH administration significantly down-regulated AQP2 and AVP gene expression. These results suggested that FH may improve the edema of NS rats by regulating the expression of AVP and AQP2 genes.

### 3.4. Effects of FH on Endogenous Metabolism of NS

Methionine is an essential amino acid and is closely related to the metabolism of various sulfur compounds in organisms. It is also involved in the synthesis of cysteine and homocysteine [38]. Studies have shown that homocysteine is mainly translocated in the kidney. Increased homocysteine levels may trigger inflammation leading to the development of kidney disease [39]. The levels of L-methionine and cysteine were significantly increased in the M group, and L-methionine was positively correlated with IL-6. L-methionine is a key metabolite of cysteine and methionine metabolism. These results suggested that the dysregulation of cysteine and methionine metabolism may be closely related to the increase in proinflammatory cytokines. FH significantly down-regulated the metabolism of cysteine and methionine and decreased the concentration of IL-6 after administration. Multi-attribute comprehensive index analysis showed that the effect value of FH on methionine and cysteine metabolism was better than that of FJ or HQ. These results suggested that FH may alleviate inflammation in rats with NS by regulating methionine and cysteine metabolism.

The product of aspartate and glutamate metabolism is succinic acid, which is a key substrate of the TCA cycle [40]. The TCA cycle is the most important way to receive energy. Disturbance of energy metabolism in the kidney may cause cell damage. This can lead to kidney damage, such as the disruption of the glomerular filtration barrier [41]. When the barrier is disrupted, the glomerular filtration membrane becomes more permeable. The entry of macromolecular proteins into the mesangial and renal tubules leads to an increase in urinary protein. After administration, FH significantly increased the contents of succinic acid, L-aspartate, and citric acid in serum, and significantly decreased BUN, Cys C, and urinary protein. Succinic acid and L-aspartate were negatively correlated with urinary protein, Cys C, and BUN, while citric acid was negatively correlated with BUN. In addition, FH significantly regulated glomerular filtration. Moreover, FH has a better regulatory effect on TCA than FJ or HQ. These results suggested that FH may reduce glomerular filtration disorders and proteinuria in rats with NS by regulating TCA cycle, aspartate, and glutamate metabolism.

## 4. Materials and Methods

### 4.1. Ethics Statement

All purchase and operation of experimental animals must be approved by the Animal Experiment Ethics Committee of Anhui University of Chinese Medicine. All experiments were conducted in accordance with the Regulations on the Management of Laboratory Animals issued by the State Science and Technology Commission.

### 4.2. Chemicals

Methoxyamine hydride (90663189), n-methyl-n-trimethylsilica-trifluoroacetamide (MSTFA, G35006), and pyridine (20170104) were purchased from Shandong West Asia Chemical Co., LTD (Linyi, China). Urease (EK170062) was supplied by Yi Ka Co., LTD (Suzhou, China). Chloroform (20170321) and methanol (20170905) were purchased from Sinopsin Chemical Research Co., LTD. (Beijing, China). All other chemicals are analytical grade and were operated according to the instructions for use. FJ, HQ, and Huangkui Capsules were purchased from the pharmacy of the First Affiliated Hospital of Anhui University of Chinese Medicine and certified by Liu Shoujin (Anhui College of Chinese Medicine, Hefei, China). IL-13 ELISA kit (96T) of rat (KS18552) was purchased from Ke Shun Science & Technology Co., Ltd. (Shanghai, China). Expressions of Bax, cleaved-caspase3, Bcl-2, STAT6, and p-STAT6 were detected using Western blot by Delph Biotechnology Co., Ltd. (Hefei, China).

### 4.3. FJ, HQ and FH Preparations

According to the dosage of FJ and HQ recorded in Shi Jin Mo’s Prescription for Medicine, the following steps were adopted to obtain FH concentrate according to the traditional decocting method: FJ (12 g) and HQ (15 g) were blended together, soaked in tap water for 30 min, then fried twice on high heat and then on low heat (1:10, *w*/*v* and 1:8, *w*/*v*), and simmered for 20 min each time. The solution was then filtered through two layers of gauze, combined, and concentrated to a concentration of 1 g/mL. FJ concentrate was prepared in the same manner as FH, without HQ, and concentrated to 0.44 g/mL. HQ concentrate was prepared in the same manner as FH, without FJ, and concentrated to 0.55 g/mL [42]. FJ, HQ, and FH concentrates were stored at 4 °C and heated before use. 

Then, 1 mL of the above samples was taken into the centrifugal tube, and after adding methanol 3 mL, was swirled for 30 s and centrifuged at 12,000 rpm. The supernatant was filtered with 0.22 μm polytetrafluoroethylene membrane. According to the provisions of the Pharmacopoeia of the People’s Republic of China (2020 edition), the corresponding index ingredients of tetrandrine, fangchinoline, and calycosin-7-glucoside were tested.

A mixed reference solution containing tetrandrine 6.14 μg, fangchinoline 6.06 μg, and calycosin-7-glucoside 1.51 μg per 1 mL was prepared by accurately weighing appropriate amounts of tetrandrine, fangchinoline, and calycosin-7-glucoside.

Ultra-high performance liquid chromatography (UPLC) analysis was performed using the Waters ACQUITY UPLC^®^H-Class system (Waters Corporation, Milford, MA, USA). C18 reversed phase column (Luna Omega Polar 2.1 × 100 mm, 1.6 μm) was used for separation. Injection volume: 2 μL; column oven temperature: 30 °C; detection wavelength: 270 nm; flow rate: 0.20 mL/min. The mobile phase consisted of methanol (A) −0.1% *v*/*v* phosphoric acid in water (B). The gradient elution procedure was as follows: 0–5 min, 10–60% A; 5–10 min, 60–80% A; 10–12 min, 80–100% A; 12–15 min, 100–10% A; 15–18 min, 10% A. The measurements were repeated to confirm the results.

The UPLC-based chromatography profiles have been provided for concentrates of FJ, HQ, FH, and reference standards of tetrandrine, fangchinoline, and calycosin-7-glucoside (Figure S4).

### 4.4. Establishment on Animal Model of NS

SD rats were randomly divided into 6 groups, including control group (K), model group (M), FJ group, HQ group, FH group, and Huangkui capsule group (Y). There were 6 rats in each group. The NS model was prepared by a single injection of adriamycin (6 mg/kg) through a caudal vein. Rats in group K were injected with the same volume of normal saline as control. The successful establishment of the nephrotic syndrome model was confirmed by the detection of a large amount of albuminuria in the urine samples of rats in M group [43]. The M group was further divided into a M (*n* = 6), FJ (*n* = 6), HQ (*n* = 6), FH (*n* = 6), and Y (*n* = 6). From the 15 days, the FJ, HQ, FH, and Y groups were given intragastric administration of FJ (14 g/kg) [14 g crude drugs per 1 kg rat], HQ (14 g/kg), FH (14 g/kg), and Huangkui capsule (1 g/kg), while the rats in group K and group M were administrated with the same volume of normal saline. The dose of each group was calculated according to the adult drug conversion formula, the specific calculation formula was as follows: every kilogram body weight dose (g/kg) dB = dA × RB/RA × (W_A_/W_B_) ^1/3^; R: specific gravity coefficient; B: Rat; A: human [44]. Among them, the dosage of FJ, HQ, and FH was determined according to the dose of FHT (14 g crude drug/kg) in previous pharmacological studies [9]. All the rats were allowed free diet of food and water during the experiment, which lasted 49 days.

### 4.5. Western Blotting Assay

The kidney tissues of the K, M, FJ, HQ, FH, and Y groups were collected using a cell scraper. Total protein was extracted from renal tissues. Protein samples (20 μg) were separated on 10% or 12% SDS polyacrylamide gel under electrophoresis and then transferred to PVDF membrane. Thereafter, the PVDF membranes were hybridized in blocking buffer overnight at 4 °C together with primary antibodies, including p-STAT6, STAT6, cleaved-caspase3, Bax, and Bcl-2 [27]. On the second day, the membranes were rinsed with PBS for 8 min × 5 times and then detected by incubation with a secondary antibody for 2 h at room temperature. It was douched with PBS for 7 min × 6 times before exposure. ECL chemiluminescence solution combined a Tanon 4500 System was used to visualize and image the membranes. In this experiment, beta-actin was used as the loading control. All proteins expression were analyzed as described.

### 4.6. Efficacy Evaluation of FJ, HQ, and FH in Rats with NS

General morphological observation, histopathology analysis of the kidneys, blood urea nitrogen, triglycerides, cholesterol, urinary protein, Cys C, TNF-α, and IL-6 were chosen as efficacy evaluation indicators.

#### 4.6.1. Histopathology Analysis

The removed kidney tissue was fixed with 10% neutral buffer formalin. Paraffin wax was used to embed dehydrated kidney tissue specimens. The Leica model RM2135 Rotary microtome (Leica, Nussloch, Germany) was used to cut and prepare 6 fixed embedded tissues into micron sections, which were stained with hematoxylin and eosin.

#### 4.6.2. BUN, Triglycerides, Cholesterol, Cys C, TNF-α, IL-6, Urinary Protein, AQP2 Gene, AQP2 Protein, AVP Gene, AVP Protein Detection

Phenethylamine turbidimetric method was used to determine the total urine protein excretion at 24 h after administration. The kits were used to determine serum triglyceride, cholesterol, Cys C, TNF-α, IL-6, and BUN levels. The expression of AQP2 gene, AQP2 protein, AVP gene, AVP protein were measured by Hefei DRP bio-tech company.

### 4.7. Serum and Urine Samples Collection

On day 60 after adriamycin was injected intravenously, 24 h urine of rats in different groups was collected by metabolic cage. Urine samples were collected and centrifuged immediately at 4 °C at a speed of 3000 rpm for 10 min. The supernatant was stored in a −80 °C refrigerator. Rats were anesthetized by intraperitoneal injection of 1% sodium pentobarbital (50 mg/kg), and blood was collected from the abdominal aorta. After being placed at room temperature for 3 h, the blood sample was centrifuged at 3000 rpm for 10 min, and the supernatant was collected and stored at −80 °C.

### 4.8. Serum and Urine Sample Preparation

In order to ensure that the endogenous components in serum and urine could be detected as much as possible by the GC-MS instrument, this study adopted a chemically derived sample pretreatment method. First, 100 µL of thawed urine was taken and 10 µL of urease (80 mg/mL) was added. The solution was mixed, gently shaken and incubated at 37 °C for 1 h. Next, 350 µL methanol: chloroform (3:1, *v*/*v*) was added and the sample was vortex treated for 10 s. A 100 µL defrosted serum sample was taken and mixed with 350 µL methanol. The samples were swirled for 10 s, and then the serum and urine samples were chemically derived as follows: the vortex urine and serum samples were centrifuged at 12,000 rpm for 10 min at 4 °C, the supernatant was taken into a 1.5 mL centrifuge tube, and nitrogen was blown dry. Then, 80 μL methxamine hydrochloride (20 mg/mL pyridine) was added, gently shaken and mixed, and incubated at 37 °C for 2 h. Then, 100 µL BSTFA (1% TMCS, *v*/*v*) was added, the tube was shaken and incubated at 70 °C for 1 h. The derived samples were cooled to room temperature and analyzed by GC-MS.

### 4.9. GC–MS Analysis

The gas chromatography–mass spectrometry system was mainly composed of Bruker 45X gas chromatograph system and SCION single quadrupole mass spectrometer (Bruker, Massachusetts, USA). DB5MS capillary column (inner diameter 30.0 × 250 µm, film thickness 0.25 µm) in the gas chromatography system, gradient programmed temperature rise conditions were as follows: The initial oven temperature was 50 °C, which was increased to 100 °C at the rate of 8 °C/min, and then the temperature was increased successively to 120, 180, 200, 250, and finally to 280 °C at the alternating rate of 4 °C and 8 °C/min. The mass spectrum conditions were as follows: helium was used as carrier gas, the flow rate was 1.0 mL/min, the current mode was constant, and the ejector shunt ratio was set to 20:1. Injector temperature 279 °C, ion source temperature 219 °C, MS temperature 219 °C. In electron ionization mode, the energy is −70 eV. Mass spectrum data were collected in full scan mode with mass/charge ratio (*m*/*z*) ranging from 50–500. In this study, quality control samples (qc) were prepared to further monitor the repeatability and stability of the method to ensure the accuracy of the experimental data. Quality control samples were prepared by mixing equal amounts of samples from each serum or urine sample. QC samples were tested every 8 samples throughout the testing process.

### 4.10. Data Processing and Statistical Analysis

The proteowizard software was used to convert original files of serum and urine samples into Net.CDF format. The XCMS1 system was used for on-line peak identification and peak comparison of gas chromatography, and the data matrix composed of *m*/*z* value, retention time, ion fragment, and peak area was obtained. Normalized data were imported into SIMCA 14.0 (Umetrics AB, Umea, Sweden) for multivariate statistical analysis. Principal component analysis (PCA) was used to reveal the overall differences between groups. The model was validated using PLS-DA and OPLS-DA. VIP > 1 and *p* < 0.05 were selected as screening criteria for differential metabolites. The metabolites were identified by similarity search in NIST11. L Mass spectrometry Library (National Institute of Standards and Technology, Gaithersburg, MD, United States) was used for the study of metabolites with NIST matching factor ≥ 700 [45].

According to the multi-attribute comprehensive index method [17], the efficacy indexes and the metabolites enriched in the metabolic pathway were treated separately, and the effect values of FJ, HQ, and FH were calculated. First, each index data (the level of efficacy indicators and response intensity of metabolites) was standardized. When the corresponding index value (*V*) of the M group was higher than that of the K group, the following formula was used (take the FH group as an example): *V_standard_* = (*V_M_*–*V_FH_*)/*V_M_*. On the contrary, the following formula was used: *V_standard_* = (*V_FH_*–*V_M_*)/*V_M_*. Then, related studies from the past 5 years were searched using the China National Knowledge Infrastructure (CNKI) and PubMed with keywords of NS related to each efficacy index, metabolite, and metabolic pathway. All indexes of rats in each group were imported into SIMCA 14.0 to calculate the VIP values of each index, and the VIP values of each index were analyzed by the PLS method using the same software. The importance of each indicator for classification was assessed according to the VIP value. Generally, an indicator with VIP > 1 was considered to have a greater contribution to classification. Finally, the weight coefficients of each indicator were comprehensively given according to the intuitive VIP processing results and the number of studies in the literature. The effect values of each efficacy index, metabolic pathway, and total metabolism were the sum of the standard value of each index multiplied by the weight coefficient. It is innovative and more comprehensive to evaluate the impact of FH on NS by the efficacy value, metabolic pathway effect value, and total metabolic effect value of the multi-attribute comprehensive index method.

## 5. Conclusions

In conclusion, FH may reduce serum cholesterol, triglyceride, IL-6 levels, and AQP2 and AVP gene expression by regulating the TCA cycle, aspartate and glutamate metabolism, and methionine and cysteine metabolism. It may inhibit IL-13/STAT6 signal pathway, regulate lipid metabolism and water-fluid metabolism, inhibit inflammation and apoptosis, and thus improve adriamycin-induced NS. In this study, the pathologic mechanism of NS was preliminarily explored by combining metabolomics and efficacy, associating efficacy indicators with endogenous substances. On the other hand, multi-attribute comprehensive index analysis was used to analyze the level of efficacy and overall metabolic changes. By comparing the differences of the FH with single herbs, the mechanism of synergistic effect of FH on the improvement of NS was preliminarily expounded. Combined with the experimental results, we speculated that FH has a better effect on NS because it has more targets in endogenous metabolism, inflammation, edema, and lipid metabolism, and the effect is more significant. The reason may be related to the synergistic effect of herbal pair on multi-components, but further exploration is needed. The research model and method adopted in this paper provide a reasonable way to explore the pathological mechanism of NS. It is of great significance to explore the compatibility theory of TCM.

## Figures and Tables

**Table 1:** Difference in potential biomarkers in groups of control group rats, nephrotic syndrome rats, and rats treated with FJ, HQ, and FH.

NO	RT (min)	Endogenous Metabolites	VIP	*p*	K-M	M-FJ	M-HQ	M-FH
S1	3.427	Cysteine	1.87	0.01	↑	↓	↓	↓
S2	3.515	Lactic acid	1.04	0.04	↑	↑	↑	↑
S3	3.757	L-methionine	1.38	0	↑	↓	↓	↓
S4	3.760	L-isoleucine	2.31	0.03	↑	↓	↓	↓
S5	3.764	Valine	2.38	0.03	↑	↓	↓	↓
S6	3.764	Glycine	2.74	0.04	↑	↓	↓	↓
S7	5.075	Citric acid	1.25	0.02	↓	↓	↓	↑
S8	5.078	Pyruvic acid	2.24	0	↓	-	-	↑
S9	16.390	Succinic acid	2.19	0	↓	↑	↑	↑
S10	18.151	L-aspartic acid	1.63	0	↓	↑	↑	↑
S11	18.156	Lactose	1.49	0	↓	↑	↑	↑
S12	18.955	Maltose	1.15	0	↑	↑	↓	↓
U1	36.635	Glycine	4.11	0.01	↓	↑	↑	↑
U2	36.621	Pyruvic acid	1.38	0.02	↓	↑	↑	↑
U3	36.628	Glutaric acid	1.92	0.02	↓	↑	↑	↑
U4	36.617	Adipic acid	1.11	0.02	↓	↓	↓	↑
U5	36.617	Uric acid	2.9	0.02	↓	↓	↓	↑
U6	36.853	4-Pyridoxic acid	4.96	0.01	↓	↑	↑	↑

## Data Availability

Data is contained within the article and Supplementary Material.

## Supplementary Materials

The following supporting information can be downloaded at: https://www.mdpi.com/article/10.3390/ph16010088/s1, Figure S1: VIP values of nephrotic syndrome related indicators. Note: Lipid metabolism index (A), glomerular filtration rate (B), edema index (C), inflammatory cell apoptosis index (D); Figure S2: Representative GC-MS TIC maps in rat blood and urine. Note: Control (K), model (M), quality control (QC), FJ, HQ and FH; Figure S3: VIP values of nephrotic syndrome-related metabolites and metabolic pathways in serum samples. Note: the VIP value of indexes of TCA cycle (A), cysteine and methionine metabolism (B), alanine, aspartate, and glutamate metabolism (C), pyruvate metabolism (D), glycine, serine and threonine metabolism (E), glutathione metabolism (F), glyoxylate and dicarboxylate metabolism (G), and total metabolism (H) was shown; Figure S4: High performance liquid chromatography of FJ, HQ, FH extract and reference of tetrandrine, fangchinoline, calycosin-7-glucoside. Table S1: Weight coefficient of related indexes of nephrotic syndrome; Table S2: Weight coefficients of indexes related to serum metabolic pathways in nephrotic syndrome.
