# Supplementary material for: Metabolomic Analysis of Stephania tetrandra–Astragalus membranaceus Herbal Pair-Improving Nephrotic Syndrome Identifies Activation of IL-13/STAT6 Signaling Pathway
Source: Pharmaceuticals (Basel). 2023 Jan 8;16(1):88. doi: 10.3390/ph16010088 (PMC9863900; doi:10.3390/ph16010088)
Supplement: Supplementary file 1 [file pharmaceuticals-16-00088-s001.zip › pharmaceuticals-2098627-Supplementary.pdf]

Table S1. Weight coefficient of related indexes of nephrotic syndrome.

Manifestations of nephrotic syndrome	Index	Weight
Lipid metabolism	T-CHO	3
	TG	2
Glomerular filtration rate	Urinary protein	3
	BUN	2
	Cys c	1
Edema	AQP gene	3
	AVP gene	3
	AQP2 protein	3
	AVP protein	1
Inflammatory cell apoptosis	IL-13	3
	TNF-α	3
	IL-6	3
	p-stat6	2
	Bcl-2	2
	Bax	1
	stat6	1
	Cleaved-caspase3	1

Table S2. Weight coefficients of indexes related to serum metabolic pathways in nephrotic syndrome

Metabolic pathway	Index	Weight
Alanine, aspartate and glutamate metabolism	Pyruvic acid	3
	Succinic acid	3
	Citric acid	2
	L-aspartic acid	1
Citrate cycle	Pyruvic acid	3
	Succinic acid	2
	Citric acid	1
Glyoxylate and dicarboxylate metabolism	Pyruvic acid	3
	Citric acid	2
	Glycine	1
Cysteine and methionine metabolism	Pyruvic acid	3
	Cysteine	3
	L-methionine	1
Glycine, serine and threonine metabolism	Pyruvic acid	3
	Cysteine	3
	Glycine	1
Pyruvate metabolism	Pyruvic acid	3
	Lactic acid	2
Glycolysis / Gluconeogenesis	Pyruvic acid	3
	Lactic acid	2
Glutathione metabolism	Cysteine	3
	Glycine	1
Primary bile acid biosynthesis	Glycine	3
Total metabolism	Glutathione metabolism	3
	Pyruvate metabolism	3
	Alanine, aspartate and glutamate metabolism	3
	Glycolysis / Gluconeogenesis	2
	Cysteine and methionine metabolism	2
	Glyoxylate and dicarboxylate metabolism	2
	Citrate cycle (TCA cycle)	2
	Primary bile acid biosynthesis	1
	Glycine, serine and threonine metabolism	1

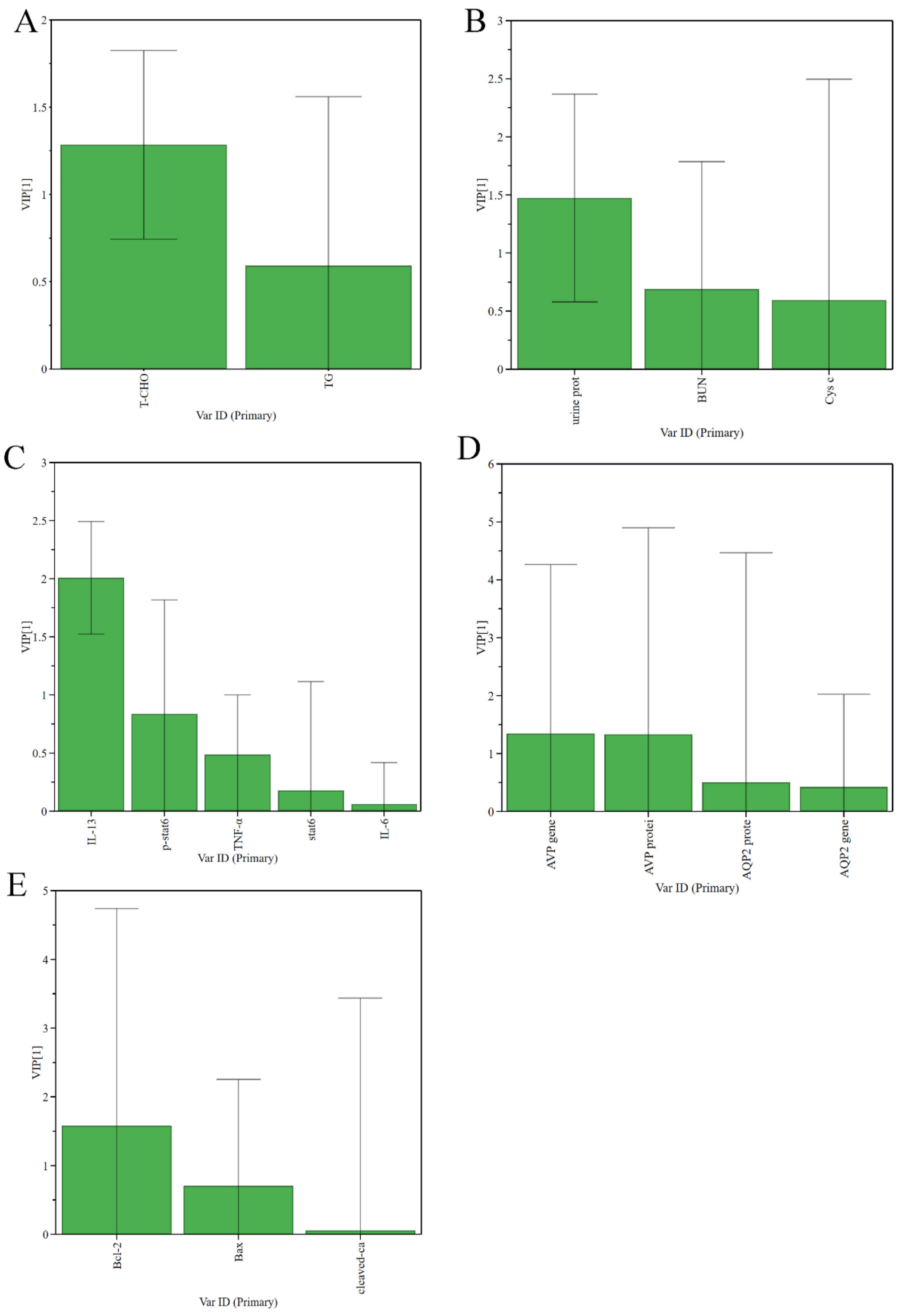

Figure S1. VIP values of nephrotic syndrome related indicators. Note: Lipid metabolism index (A), glomerular filtration rate (B), edema index (C), inflammatory cell apoptosis index (D).

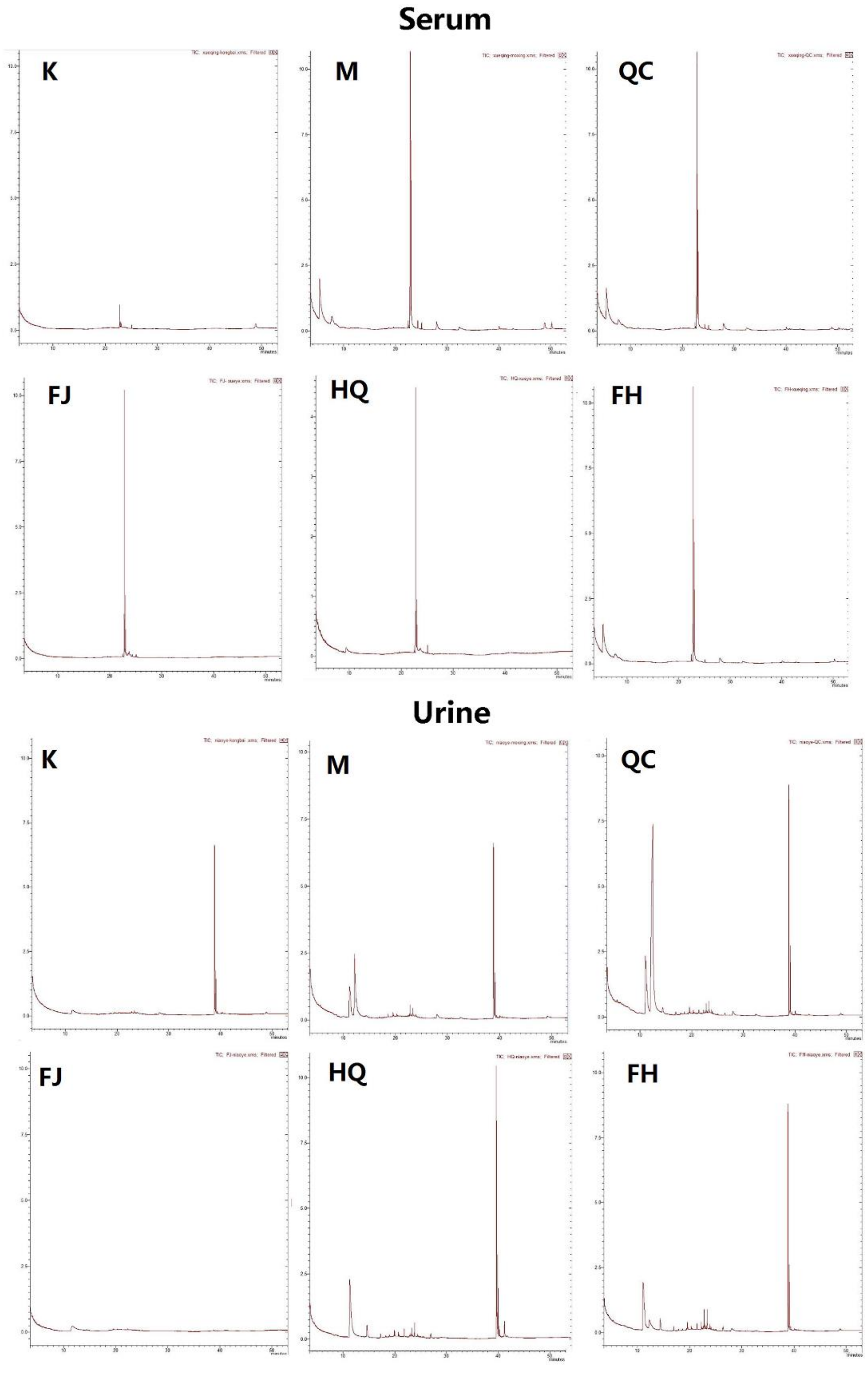

Figure S2. Representative GC-MS TIC maps in rat blood and urine. Note: Control (K), model (M), quality control (QC), FJ, HQ and FH.

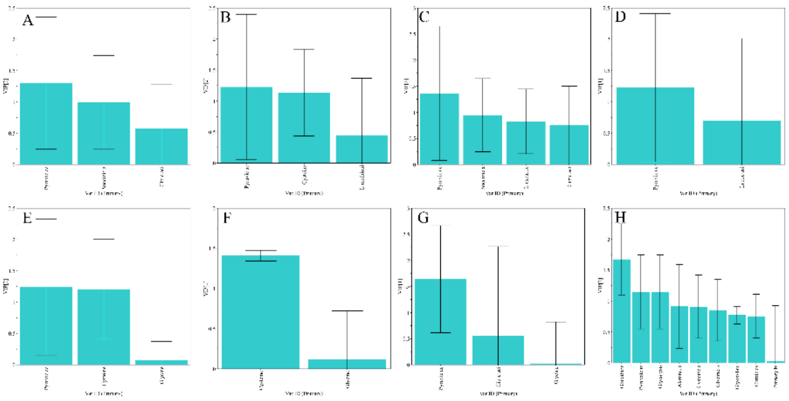

Figure S3. VIP values of nephrotic syndrome-related metabolites and metabolic pathways in serum samples. Note: the VIP value of indexes of TCA cycle (A), cysteine and methionine metabolism (B), alanine, aspartate, and glutamate metabolism (C), pyruvate metabolism (D), glycine, serine and threonine metabolism (E), glutathione metabolism (F), glyoxylate and dicarboxylate metabolism (G), and total metabolism (H) was shown.

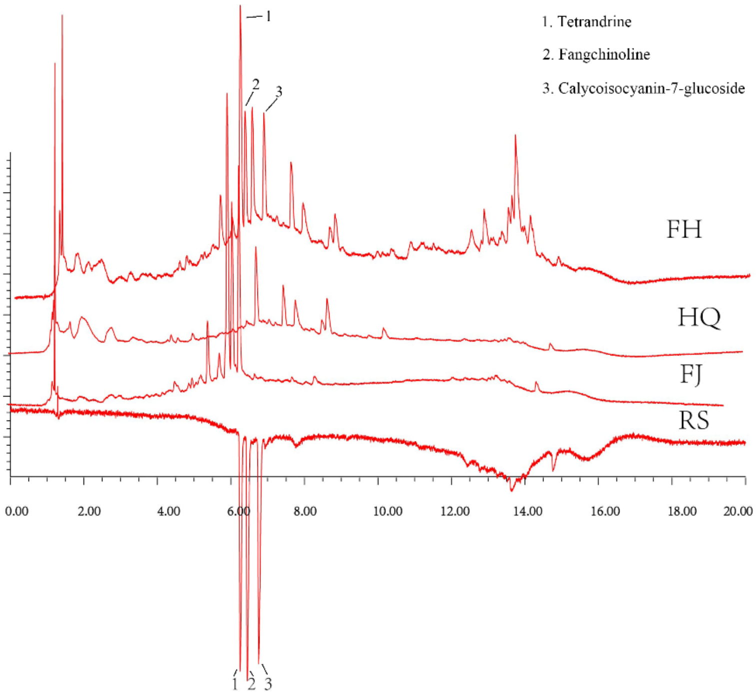

Figure S4. High performance liquid chromatography of FJ, HQ, FH extract and reference of tetrandrine, fangchinoline, calycosin-7-glucoside.
